# Items

**Published:** 1882-06

**Authors:** 


### Items.

---

THIS spring has been marked by an unusual mortality among the notable men of the medical profession in the United States. Dr. James R. Wood, long identified with the medical profession and with medical teaching in New York city, died after an illness of only four days, from double pneumonia, May 4th, in the sixty-sixth year of his age.

Dr. Joseph Pancoast, of the Jefferson Medical College, died at his residence in Philadelphia, from congestion of the lungs, Tuesday, March 9th, at an advanced age. He filled a medical teachers chair for more than forty years. Dr. Pancoast was one of the oldest and wealthiest citizens of Philadelphia. It is said that his estate is valued at one million dollars.

Dr. John T. Hogden, of St. Louis, recently President of the American Medical Association, died after a few hours illness, April 28th. He was subject to sudden obscure attacks similar to the last which proved so promptly fatal. The post-mortem revealed a peculiar obstruction of the gall duct, resembling a stricture, by a valvular fold of the lining membrane; no calculi, but evidences of recent peritonitis and remnants of old and repeated attacks were found.

Dr. C. B White, of New Orleans, late President of the American Public Health Association, died April 22, in the fifty-seventh year of his age. Dr. White was a laborious student, an enthusiastic hygienist, a useful man. He had been in feeble health for a long time and his death was not unexpected, but it has nevertheless caused a vacancy that cannot be easily filled.

---

Referring to the refusal of the Philadelphia County Medical Society to admit women to membership, the *Boston Medical and Surgical Journal* pertinently says: "The advocates of equal rights, though defeated, are but little dismayed by this result, which was not unexpected; the mere fact that the names of women candidates have been allowed by the board of censors to be presented for balloting is evidence of marked change, as contrasted with the time when men were professionally ostracised for teaching in the Women's College, or for simply consulting with female practitioners. Now there is apparent among some of our principal practitioners much warm rivalry as to who will bid highest for the consulting practice with the weaker vessels, just as in a neighboring community attention has been called recently to a similar break-neck race for the homœopathic and irregular constituency. Such men do exist in the profession even in Philadelphia, but they have not yet succeeded in capturing our medical societies."

---

PHOTOGRAPHY AND THE INTERNATIONAL MEDICAL CONGRESS.—*The Medical Press and Circular* says: "It is fitting that the greatest congress of physicians, surgeons and men of science which the world ever saw should be recorded in art as well as in literature and that not only its work but that its *personel* should be suitably commemorated." The art record, it testifies, has been produced as creditable to the congress itself as to the artists and publishers who undertook the task. It is a splendid work of permanent carbon photography.

Six hundred and eighty-four members from all parts of the world are included in the picture, as they sat in the great Gothic Hall, a room of imposing architectural proportions.

The picture is published in two sizes, 30 by 47 inches and 20 by 29 inches. The likenesses are said to be excellent and the picture, as a whole, very handsome.

---

THE CONTRACT for publishing the new edition of the Pharmacopœia has been awarded to Messrs. William Wood & Company, the well known publishers of New York, by a sub-committee of the committee on revision. Messrs. Wood & Co. offered, in the bid which was accepted, 10 per cent. copy-right and guaranteed the sale of eleven thousand copies at $4.00 each. The five other competing firms are not satisfied with the award and complain that justice has not been done and that the best available terms have not been secured.

It remains to be seen what the entire committee will do about it.

The Medical Association of Georgia and the Code.—At the thirty-third annual session of the Medical Association of Georgia, held in Atlanta, April 19–21, 1882, th e following resolutions were offered, and were enthusiastically adopted, with one dissenting voice :

"*Resolved*, That the Medical Association of Georgia, in convention assembled, re-affirms its devotion to the time-honored code of ethics of the American Medical Association, which has heretofore been its accepted chart, and shall henceforward be its trusted guide.

"*Resolved*, That this body deprecates any and all attempts on the part of medical organizations to break down this honorable barrier between the regular medical profession and the vast and varied domain of quackery ; and do earnestly protest against any departure from the true spirit of the supreme law of the organized profession of the union.

"*Resolved*, That a duly attested copy of these resolutions be presented by the delegates from this body to the American Medical Association, in open session, at its approaching meeting."

---

The New Code (?).—The open letter of Dr. W. O. Baldwin (*American Medical Weekly*), a former President of the American Medical Association, to Dr. Lewis a Sayre, will be read with peculiar interest and satisfaction. The manner in which he demolishes the specious claim made by the framers of the New York Code of Medical Ethics, and of those who have garbled this to sustain their unsustainable and disreputable position, is very thorough and happy. He also quotes from the transactions of the Association of 1869 to show that this body, having anticipated some such claim as is now made in regard to consultation with "legally qualified" but irregular practitioners, unanimously denounced all such consultations and placed those thus engaged beyond the pale of professional recognition. This "letter" is sent to the entire medical press of the country, that the reading of it and the comments upon it may be completed prior to the meeting of the American Medical Association, at St. Paul.

Small-pox in Atlanta.—Many absurd stories have gone abroad concerning the existence of small-pox in this city, and the acceptance of mere rumors as solid facts by a few neighboring villages has produced an unnecessary but a very amusing amount of scare. The disease, since its advent here, has not averaged one and a half cases a day. There never has been in any city a more thorough system of vaccination or a more rigid and effective system of inspection. It is silly, under these circumstances, to talk about an epidemic.

---

It now appears that the country is to be favored with two colleges for medical practitioners. One is already organized and announced at St. Louis, and the other will be located in New York. It is intimated that the post-graduate faculty of the medical department of the University of New York, who resigned their connection with the University school very recently, are to compose the faculty of the new departure. It is more than likely that some of the members, at least, of the late faculty will be actively engaged in the movement.

---

The American Association for the Advancement of Science will hold its thirty-first meeting in Montreal, Canada, commencing on Wednesday, August 23rd. J. W. Dawson, of Montreal, is President and F. W. Putnam, Salem, Mass., is the permanent secretary.

---

And now it is in order for the homœopaths, eclectics, root, steam and cancer doctors, and the balance of this prolific class, to rise up and refuse to recognize or consult with the New York "regulars."

---

The American Medical Association will convene in its thirty-third annual session at St. Paul, Minnesota, Tuesday, June 6th, and continue four days.

---

Charles Darwin, the celebrated naturalist and biologist is dead.

